# Roles of lncRNAs Mediating Wnt/β-Catenin Signaling in HCC

**DOI:** 10.3389/fonc.2022.831366

**Published:** 2022-03-09

**Authors:** Yating Xu, Xiao Yu, Zongzong Sun, Yuting He, Wenzhi Guo

**Affiliations:** ^1^ Department of Hepatobiliary and Pancreatic Surgery, The First Affiliated Hospital of Zhengzhou University, Zhengzhou, China; ^2^ Key Laboratory of Hepatobiliary and Pancreatic Surgery and Digestive Organ Transplantation of Henan Province, The First Affiliated Hospital of Zhengzhou University, Zhengzhou, China; ^3^ Open and Key Laboratory of Hepatobiliary and Pancreatic Surgery and Digestive Organ Transplantation at Henan Universities, Zhengzhou, China; ^4^ Zhengzhou Key Laboratory of Hepatobiliary & Pancreatic Diseases and Organ Transplantation Medicine, Zhengzhou, China; ^5^ Department of Obstetrics and Gynecology, The Third Affiliated Hospital of Zhengzhou University, Zhengzhou, China

**Keywords:** hepatocellular carcinoma, long noncoding RNA, Wnt pathway, biomarker, prognosis

## Abstract

Hepatocellular carcinoma (HCC) is considered the second most deadly cancer worldwide. Due to the absence of early diagnostic markers and effective therapeutic approaches, distant metastasis and increasing recurrence rates are major difficulties in the clinical treatment of HCC. Further understanding of its pathogenesis has become an urgent goal in HCC research. Recently, abnormal expression of long noncoding RNAs (lncRNAs) was identified as a vital regulator involved in the initiation and development of HCC. Activation of the Wnt/β-catenin pathway has been reported to obviously impact cell proliferation, invasion, and migration of HCC. This article reviews specific interactions, significant mechanisms and molecules related to HCC initiation and progression to provide promising strategies for treatment.

## Introduction

Liver cancer has become one of most prevalent malignant diseases worldwide, and hepatocellular carcinoma (HCC) accounts for most liver cancer cases (1–3). Owing to the increasing incidence and mortality, HCC is considered the second most deadly cancer worldwide (4). As clinical research is rapidly advancing, the short survival time and rising recurrence rate of HCC present difficulties in patient treatment (5–7). Thus, deeply exploring the pathogenesis mechanism could remarkably improve prognosis (8–10). HCC generally develops from chronic liver diseases commonly caused by infection with hepatitis B virus (HBV) and hepatitis C virus (HCV) (11–13), and other pathogenic factors also play important roles in the initiation of malignancy. Recently, emerging investigations have concentrated on the molecular mechanisms by which long noncoding RNAs (lncRNAs) regulate HCC tumorigenesis and progression by affecting Wnt/β-catenin signaling (14–16).

As diverse RNA detection methods are rapidly improved and widely applied, a large number of noncoding RNAs have been discovered and initially referred to in broad studies (17–19). LncRNAs (Long non-coding RNAs) are a special category of noncoding RNAs with no less than 200 nucleotides. Accumulating evidence has proven and emphasized the indispensable role of lncRNAs that participate in the pathogenesis of multiple cancers (20–22). For example, it was found that lncRNA HCG11 (human leukocyte antigen complex group 11) could enhance the expression of GFI1 (growth factor independence-1) to suppress the proliferation and invasion of cancer cells, functioning as a regulator of miR-942-5p *via* sponging in cervical cancer (23). Thus, lncRNA HCG11 is considered a vital suppressor of HCC. In addition, lncRNA DDX1-AS1 was confirmed to regulate HCC cell proliferation and has a strong correlation with a poor prognosis. It was reported that demethylation in HCC could increase the expression of DDX1-AS1 through the inhibition of P53 in the poly(ADP-ribose) polymerase 1 (PARP1)–p53 axis (24).

The Wnt/β-catenin pathway, an evolutionarily conserved signaling axis and a complicated protein network, plays a pivotal role in governing numerous physiological processes, including proliferation, differentiation, and tissue homeostasis (25–27). A previous study uncovered that dysregulation of the Wnt/β-catenin cascade has a major impact on the complicated developmental process of multiple cancers (28–30). For instance, overexpression of lncRNAs positively related to the Wnt/β-catenin signaling pathway plays an indispensable role in the progression of bladder cancer (31–33). Analogously, lncRNA AB073614 is highly expressed in glioma and indicates a shorter survival time; this lncRNA targets sex-determining region Y-box 7 (SOX7) to enhance the expression of Wnt/β-catenin and promote the proliferation and metastasis of glioma cells (34). In addition, the upregulation of lncRNA SNHG1 could promote the proliferation of Non-Small-Cell Lung Carcinoma (NSCLC) cells (35). SNHG1 overexpression is negatively correlated with miR-101-3p expression, and sex-determining region Y-box 9 (SOX9) serves as the downstream effector of miR-101-3p. SOX9 is considered a vital regulator of the activation of Wnt/β-catenin signaling (36–38).

Despite increased knowledge regarding associations between lncRNAs and the Wnt/β-catenin pathway in multiple cancers, the underlying mechanisms in the occurrence and development of HCC remain unclear. Therefore, exploration of the effect of interactions with lncRNAs and the Wnt/β-catenin pathway is still a rewarding direction. In this review, we summarized and highlighted the mechanisms of lncRNAs involved in the Wnt/β-catenin pathway in HCC. We expect that these discoveries could provide prospective and creative ideas for targeted treatment for HCC patients.

## The Wnt/β-Catenin Pathway Is Related to HCC

A previous report confirmed that the Wnt cascade is commonly divided into two pathways—the canonical and noncanonical signal pathways (39–41)—that mediate the biological process of cell proliferation and differentiation by affecting the transcriptional framework (42). Canonical signaling is known as the Wnt/β-catenin pathway, and the abnormal expression of β-catenin directly contributes to tumorigenesis (43–45). It was reported that destruction of β-catenin is mainly determined by the key degradation complex, which contains scaffold proteins (AXIN), the human tumor suppressor adenomatous polyposis coli (APC), glycogen synthase kinase 3β (GSK3β), and casein kinase 1 alpha 1 (CSNK1A1) (46–48). The degradation complex could lead to the phosphorylation of β-catenin at serine/threonine residues of the N-terminus; subsequently, beta-transducin repeat containing E3 ubiquitin protein (β-TRCP) can recognize and ubiquitinate phosphorylated β-catenin to facilitate its degradation in proteasome (46). Mutations of members from degradation complex presented remarkable correlation with hepatocarcinogenesis. Unphosphorylated *CTNNB1* (β-catenin gene) accumulates in the cytoplasm and accesses the nucleus, interacting with T cell-specific factor (TCF)/lymphoid enhancer-binding factor (LEF) and activating a wide range of signaling cascades (30). When *CTNNB1* dramatically accumulates in the nucleus and cytoplasm, the probability of HCC cell invasion, proliferation, and deterioration is significantly increased (49). Therefore, *CTNNB1* knockdown could block the process of migration and invasion in HCC and improve patient prognosis (50).

Although mutation of *CTNNB1* was shown to be associated with HBV-related cancer in previous research (51), other functional mechanisms of *CTNNB1* in HBV-related HCC have also gradually been revealed (52). For instance, the HBV gene positively mediates the expression of von Willebrand factor C and EGF domains (VWCE/URG11) at high levels and binds with APC to boost the activation of *CTNNB1*. In humans, nineteen Wnt ligands and ten receptors from the frizzled class receptor (FZD) family have been verified (53). Several experiments confirmed that elevated expression of Wnt1 and Wnt3a is positively associated with the HCV core protein in HCC (54, 55). The mutual effects of Wnt3a and FZD7 (Frizzled class receptor 7) can trigger the Wnt signaling pathway. FZD7 upregulation might give rise to migration in the early stage of HCC (56, 57). The mechanisms of *CTNNB1* involvement in HCV-related HCC have been explored in more detail than those of HBV-related tumors. HCV can increase miRNA-155 expression, facilitating the nuclear accumulation of *CTNNB1* and triggering the expression of downstream targets (58). Additionally, NS5A (nonstructural protein 5A), the core protein of HCV, was found to accelerate GSK3β phosphorylation and actively regulate PI3K, contributing to increased *CTNNB1* expression (54, 59, 60).

## LncRNAs Related to HCC

LncRNAs are no less than 200 nucleotides in length, and they are unable to encode proteins. According to the principle of correlative location for the closest coding genes, lncRNAs fall into the five following varieties: sense lncRNA, antisense lncRNA, intergenic lncRNA, intronic lncRNA, and bidirectional lncRNA (61–63). The distribution of lncRNAs in the nucleus or cytoplasm and the dysregulation of lncRNAs may bring about many diseases (64–66). Increasing experiments about lncRNA can offer a bright orientation of earlier diagnosis and effective treatment of HCC (67, 68).

**Figure 1:**
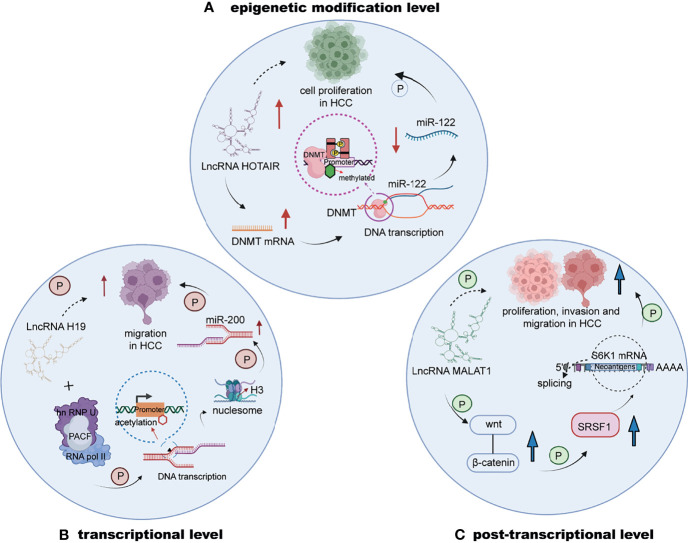
The regulation of lncRNAs in HCC at the epigenetic, transcriptional or posttranscriptional level. **(A)** LncRNA HOTAIR upregulates DNMTs to increase the methylation of the promoter of miR-122, decreasing the expression of miR-122 to stimulate HCC cell proliferation. **(B)** The interaction of lncRNA H19 and the hnRNP U/PCAF/RNA pol II complex upregulates the transcriptional expression of the miR-200 family. **(C)** LncRNA MALAT1 is highly expressed and induces the progression of HCC by triggering the Wnt pathway.

Emerging investigations have revealed that lncRNAs mainly exert epigenetic, transcriptional or posttranscriptional regulation effects on downstream factors (**Figure 1**), leading to alterations in expression and stability (69). For example, lncRNA HOTAIR (HOX transcript antisense intergenic RNA) can increase the expression of DNMTs to catalyze epigenetic methylation of the promoter region of miR-122, filled with GC islands (70, 71), which leads to the inhibition of miR-122 expression. MiR-122 downregulation might induce the abnormal expression of cyclin G1 and promote cell proliferation in HCC (72). At the transcriptional level, lncRNAs have been reported to directly mediate and alter the expression of noncoding genes, such as miRNAs. In HCC, downregulated H19 was shown to be positively correlated with migration (73). H19 directly interacts with the hnRNP U/PCAF/RNA pol II complex, promoting the transcriptional expression of the miR-200 family through the acetylation of histone H3 in the promoter region. Moreover, the posttranscriptional regulation of lncRNAs is mainly regarded as the effect of alterations in alternative splicing and mRNA stability. The lncRNA termed metastasis-associated lung adenocarcinoma transcript 1 (MALAT1) is highly expressed in HCC and triggers the Wnt pathway, and cells with MALAT1 upregulation tend to exhibit increased splicing factor serine and arginine rich splicing factor 1 (SRSF1) expression. The mentioned effect can also induce apoptosis by alternatively splicing of S6 kinase 1 (S6 K1), further increasing the expression of the mTOR pathway to affect HCC progression (74). A number of lncRNAs also regulate downstream factors and affect important pathways involved in vital biological processes of HCC.

## The lncRNA/Wnt/β-Catenin Axis in HCC

In emerging studies, a number of molecules were considered to serve as transcriptional regulators or directly mediate gene expression, participating in the regulation of the Wnt/β-catenin pathway by affecting the expression of several lncRNAs (75). It was confirmed that β-catenin-specific proteins were key regulators that triggered the Wnt/β-catenin cascade (76). Recently, the study of the involvement of the lncRNA/Wnt/β-catenin axis in the pathogenesis of malignant tumors has been successful (77, 78). We conclude that the primary lncRNA/Wnt/β-catenin axis plays a role in HCC (**Table 1**).

**Table 1:** Expression of lncRNAs in related lncRNA/Wnt/β-catenin axes in HCC.

LncRNA	Expression	Related regulation axis	Prognosis	Diagnosis value	References
CRNDE	high	CRNDE/Wnt2/Frizzled4/Wnt/β-catenin axis	poor	profitable	(79, 80)
LINC00346	high	LINC00346/miR-542-3p/FZD7/Wnt/β-catenin axis	poor	profitable	(81)
DUXAP10	high	DUXAP10/Wnt/β-catenin	poor	/	(82)
SOX9-AS1	high	SOX9-AS1/SOX9/miR-5590-3p/Wnt/β-catenin axis	poor	profitable	(83)
MiR143HG	down	MiR143HG/Wnt/β-catenin	favorable	/	(84)
lncRNA-CR594175	high	lncRNA-CR594175/Wnt/β-catenin	poor	profitable	(85)
MiRNA194-2HG	high	MiRNA194-2HG/miR-1207-5p/Wnt/β-catenin	poor	/	(86)
FEZF1-AS1	high	FEZF1-AS1/miR-107/Wnt/β-catenin	poor	profitable	(87)
HOTAIR	high	HOTAIR/miR-34a/Wnt/β-catenin	poor	/	(88)
LINC00210	high	LINC00210/Wnt/β-catenin	poor	profitable	(89)
LncRNA TCF7	high	LncRNATCF7/TCF7/Wnt/β-catenin	poor	profitable	(90)
PRR34-AS1	high	PRR34-AS1/miR-296-5p/E2F2/SOX12/Wnt/β-catenin	poor	/	(91)
H19	high	H19/EZH2/Wnt/β-catenin	poor	profitable	(92)
ANRIL	high	ANRIL/mi-RNA191/Wnt/β-catenin	poor	/	(93)
DSCR8	high	DSCR8/miR-485-5p/FZD7/Wnt/β-catenin	poor	profitable	(94)
FOXD2-AS1	high	FOXD2-AS1/EZH2/DKK1/Wnt/β-catenin	poor	/	(95)
LncRNA-NEF	down	LncRNA-NEF/Wnt/β-catenin	favorable	profitable	(96)
CASC2c	down	CASC2c/Wnt/β-catenin	favorable	/	(97)
TP53TG1	down	TP53TG1/PRDX4/Wnt/β-catenin	favorable	profitable	(87)
SUMO1P3	high	SUMO1P3/miR-320a/Wnt/β-catenin	poor	/	(98)
CASC15	high	CASC15/SOX4/Wnt/β-catenin	poor	profitable	(99)
OTUD6B-AS1	high	OTUD6B-AS1/Wnt/β-catenin	poor	profitable	(100)
LINC01278	high	LINC01278/Wnt/β-catenin	poor	profitable	(101)
LINC00662	high	LINC00662/miR-15a/miR-16/miR-107/Wnt3a/Wnt/β-catenin	poor	profitable	(102)
DGCR5	down	DGCR5/Wnt/β-catenin	favorable	profitable	(103)
CCAL	high	CCAL/AP-2α/Wnt/β-catenin	poor	profitable	(104)
OGFRP1	high	OGFRP1/Wnt/β-catenin	poor	/	(105)
SNHG5	high	SNHG5/miR-26a-5p/Wnt/β-catenin	poor	profitable	(106)
LINC01391	down	LINC01391/ICAT/Wnt/β-catenin	favorable	profitable	(107)
ASB16-AS1	high	LncRNA ASB16-AS1/miR-1827/FZD4/Wnt/β-catenin	poor	/	(108)
RUNX1-IT1	down	RUNX1-IT1/miR-632/Wnt/β-catenin	favorable	profitable	(109)
LINCROR	high	LINCROR/Wnt/β-catenin	poor	/	(110)
ANCR	down	ANCR/Wnt/β-catenin	favorable	/	(111)
LncAY	high	LncAY/YTHDF2/BMI1/Wnt/β-catenin	poor	/	(112)
LINC00355:8	high	LINC00355:8/miR-6777-3p/Wnt10b/Wnt/β-catenin	poor	profitable	(113)
DLGAP1-AS1	high	DLGAP1-AS1/miR-26a/b-5p/Wnt/β-catenin	poor	profitable	(114)

## The lncRNA CRNDE/Wnt2/Frizzled 4/Wnt/β-Catenin Axis

LncRNA colorectal neoplasia differentially expressed (CRNDE) has a tendency to be abnormally expressed in colorectal neoplasia samples (115, 116). Substantial experiments have gradually recognized the participation of CRNDE in cell proliferation, apoptosis, invasion, and migration in various malignant tumors (117–119). CRNDE expression is higher in HCC tissues than in adjacent tissues. In addition, overexpression of CRNDE could promote HCC cell proliferation, predicting a poorer prognosis in patients. Mechanistically, CRNDE is reported to upregulate the expression of cyclins and promote cell cycle transition from the G0/G1 phase to S phase (79). When CRNDE was silenced, inhibition of HCC cell proliferation, invasion, and metastasis was observed (80). Knockdown of CRNDE also reduced the expression of slug, twist, N‐cadherin, and vimentin, resulting in blockade of the oncogenic effect of epithelial to mesenchymal transition (EMT) induction. Numerous studies have demonstrated that EMT plays a vital role in invading and migrating in diverse cancers (120, 121). Activation of Wnt/β-catenin is considered an essential process in stimulating EMT progression (122). CRNDE downregulation might suppress the expression of Wnt2, Frizzled 4, and β‐catenin to affect the Wnt/β-catenin pathway. Wnt2 belongs to the WNT gene family and promotes the expression of β‐catenin to trigger the canonical Wnt pathway (123, 124). It was reported that the HCV core protein could increase the gene expression of Wnt2 in the SMMC7721 cell line (125). In addition, overexpression of Wnt2 mRNA was detected to exert an indispensable effect on disease processes, such as colorectal polyps, primary colorectal cancer, and the liver metastasis of colorectal cancer (126). In humans, nineteen Wnt ligands and ten receptors from the FZD family have been verified (51). Frizzled 4 downregulation represses the proliferation of cervical cancer cells by increasing the expression of miR-375 (127). These results indicated that high expression of CRNDE promotes the invasion of HCC cells by inducing upregulation of the Wnt/β‐catenin cascades. On the basis of the mentioned study, CRNDE is regarded as a potential therapeutic target for HCC.

## LncRNA ASB16-AS1/miR-1827/FZD4/Wnt/β-Catenin Axis

In HCC, lncRNA ASB16 antisense RNA1 (ASB16-AS1) was found to aberrantly express in high level and have positively association with unsatisfied outcome (108). On the contrary, knockdown of ASB16-AS1 could exert the anti-tumor effect on biological function of cancer cell, containing proliferation, invasion, and metastasis. Simultaneously expression of miR-1827 presented increasing after knock of ASB16-AS1, which indicated the fact that activity of miR-1827 might be controlled by lncRNA ASB16-AS1. FZD4 (Frizzled class receptor 4) is regard as the significant molecule to active Wnt/β-catenin signaling pathway (128). Upregulated miR-1827 could negatively mediated expression of FZD4 to inhibit progression of HCC. When FZD4 was high-expressed, the effect from knockdown of ASB16-AS1 would be reversed. Briefly, lncRNA ASB16-AS1 might sponge miR-1827 to decrease its expression level, resulting in upregulating FZD and triggering Wnt/β-catenin pathway to involve in tumorigenesis of HCC. However, whether lncRNA ASB16-AS1 has upstream regulators to modulate lncRNA ASB16-AS1/miR-1827/FZD4/Wnt/β-catenin axis is still unclear, and more explorations and researches are required.

## The LINC00346/miR-542-3p/FZD7/Wnt/β-Catenin Axis

LncRNA LINC00346 was is highly expressed and plays tumor-promoting roles in several tumors (129). LINC00346 was commonly verified to be highly expressed in HCC tissues. LINC00346 functions as an oncogenic regulator, boosting the viability, migration, and developmental ability of HCC cells. Mechanistically, LINC00346 attenuates the suppressive effect of miR-542-3p on WDR18 (WD repeat domain 18)) expression by sponging miR-542-3p, contributing to increased Wnt/β-catenin expression *in vitro* and *in vivo* (130–132). MiR-542-3p presented diverse effects in several studies *via* the regulation of various signaling pathways. One study confirmed that miR-542-3p could trigger the TGF-β/Smad pathway to promote the migration of HCC cells. In contrast, miR-542-3p could downregulate FZD7/Wnt to block the proliferation of HCC cells in another study (97). WDR18 was observed to be expressed predominantly in HCC tissues and regarded as an oncogenic regulator of tumorigenesis in HCC. Activation of Wnt/β-catenin signaling cascades might partly depend on WDR18. Enhancing the expression of WDR18 could promote increased β-catenin expression, triggering the Wnt/β-catenin signaling pathway (133–135). Furthermore, accumulating evidence has proven that the final downstream target of the Wnt/β-catenin cascades is MYC in ALK-positive anaplastic large cell lymphoma and basal stem cells (81). However, more molecular mechanisms remain unreported, and further exploration of Wnt/β-catenin signaling in HCC is necessary.

## The lncRNA DUXAP10/Wnt/β-Catenin Axis

LncRNA DUXAP10 was reported to be expressed at increasing levels in different malignant tumors (136), such as bladder cancer (137), prostate cancer (138), renal cell carcinoma (139), liver cancer (38) and colorectal cancer (140). Overexpression of DUXAP10 was observed in HCC tissues versus normal tissues (141, 142), which indicates that DUXAP10 is a risk predictor in patients with HCC (83). DUXAP10 overexpression is significantly correlated with the malignant behavior of HCC cells and could predict the survival time of patients with advanced-stage disease (143). Inhibition of DUXAP10 dramatically weakened the viability and proliferative ability of SMMC-7721 cells and HepG2 cells. In concordance with the result from ovarian cancer in DUXAP10. It was reported that silencing DUXAP10 caused damage to the development of lung cancer *in vivo* (144). In HCC, knockdown of DUXAP10 could mediate the promotion of apoptosis and suppression of cell proliferation. Additionally, downregulation of PI3K/Akt was affected by knockdown of DUXAP10 (140, 145). The PI3K/Akt signaling pathway was found to be involved in the process of invasion and migration as well as the Wnt/β-catenin signaling pathway. DUXAP10 is considered to regulate EMT to mediate the progression of HCC *via* PI3K/Akt and Wnt/β-catenin cascades. Thus, inhibition of DUXAP10 could strongly suppress EMT (82).

## The lncRNA SOX9-AS1*/*SOX9/miR-5590-3p/Wnt/β-Catenin Axis

SOX9 is a member of the sex-determining region Y (146) box gene (SRY) superfamily and is a transcription factor (147) that plays an essential role in gene expression regulation (148, 149). A previous study confirmed the facilitative effect of SRY members in a variety of tumors. LncRNA SOX9 antisense RNA 1 (SOX9-AS1) is regarded to promote SOX9 expression. SOX9 has a positive association with the development of HCC and commonly contributes to the dismal outcome of patients (150). In a recent study, miRNA-138 is perceived as an inhibitor that affects HCC cell growth *via* the suppression of SOX9. Additionally, SOX9 might have an impact on mediating β-catenin expression in canonical signaling and may strongly affect the expression of downstream regulators, such as cyclin D1 and c-Myc (38), to promote the development of malignancies. Activation of the Wnt/β-catenin cascades exert an indispensable effect on cell proliferation, invasion, and metastasis in HCC (141, 142). Silencing of SOX9 or SOX9-AS1 reduces Wnt/β-catenin expression, leading to the inhibition of EMT. SOX9-AS1 is highly expressed in HCC tissue, but miRNA-5590-3p is expressed at low levels. SOX9-AS1 might modulate SOX9/Wnt/β-catenin by regulating miR-5590-3p. When mutations occur at the miRNA-5590-3p site on SOX9-AS1, the activating effect of SOX9 upregulation *via* the SOX9/β-catenin axis is attenuated. These results suggest that miRNAs might regulate SOX9-AS1 *via* SOX9/miR-5590-3p/Wnt/β-catenin axis (83).

## Other lncRNAs Involved in lncRNA/Wnt/β-Catenin Axes

Lnc RNA MiR143HG is downregulated in HCC cells and commonly indicates shorter survival time in patients. A recent study shed light on the function of miR-143HG in HCC and found that miR-143HG inhibited the activation of Wnt/β-catenin cascades to restrict the proliferation of HCC cells (84). In contrast, lncRNA-CR594175 was proven to be expressed at higher levels in primary HCC than in adjacent tumor tissues. LncRNA-CR594175 is considered an effector that reverses the downregulation of *CTNNB1* from hsa-miR-142-3p, facilitating HCC progression by triggering the Wnt pathway (85). Similarly, patients with overexpression of miRNA194-2HG in HCC exhibit a poor prognosis. MiRNA194-2HG functions as a competing endogenous RNA (ceRNA) to sponge miR-1207-5p, contributing to enhanced cell invasion and migration in HCC by activating Wnt signaling (86). Another investigation found that lncRNA-TP53TG1 expression was low in HCC tissues. LncRNA-TP53TG1 expression is regarded as an independent prognostic factor in patients with HCC. Additionally, lncRNA-TP53TG1 acts as a suppressor in HCC to negatively influence several processes of cell proliferation, invasion and metastasis. The inhibition of proliferation *via* lncRNA-TP53TG1 is mediated by the alteration in the ubiquitination levels of PRDX4 (peroxiredoxin 4) protein and the activation of the Wnt pathway (151). Zhu et al. (87) revealed that lncRNA FEZF1-AS1 is upregulated in HCC and facilitates malignant behavior, such as early invasion and metastasis. The molecular mechanism showed that FEZF1-AS1 increases the development and progression of HCC through modulation of the miR-107/Wnt/β-catenin axis. A previous study indicated that miR-107 serves as a suppressor involved in a variety of cancers (152–154). In HCC, miR-107 downregulation was observed. Overexpression of miR-107 has been proven to downregulate β-catenin and wnt3a and increase the expression level of p-GSK-3β, resulting in the blockade of Wnt signaling activation. Thus, we can presume that the inhibitory lncRNA FEZF1-AS1 might interact with miRNA-107 to alter the expression of the Wnt pathway. In brief, these lncRNAs are reported to have a significant association with HCC prognosis in patients with HCC, and the mechanism by which lncRNAs regulate the Wnt/β-catenin pathway needs to be further explored. Fortunately, the mentioned study provides promising ideas for diagnostic or therapeutic approaches in HCC.

## Biological Functions of lncRNA/Wnt/β-Catenin Axes in HCC

LncRNAs impact a variety of functions of HCC through the regulation of Wnt n cascades. Thus, we summarized the mechanisms related to the clinical features of HCC in **Table 2**, expecting to find effective approaches to prolong the survival time of patients.

**Table 2:** Role and clinical functions of lncRNAs in HCC.

LncRNA	Role	Clinical functions	Related factors	References
LINC00210	oncogene	Promote development of cancer stem cells	CTNNBIP1	(89)
LncRNA TCF7	oncogene	Promote development of cancer stem cells	SWI, SNF, β-catenin	(90)
PRR34-AS1	oncogene	Promote tumorigenesis	miR-296-5p, E2F2, SOX12, β-catenin	(91)
H19	oncogene	Promote tumorigenesis	EZH2, H3K27me3, β-catenin	(92)
ANRIL	oncogene	Promote proliferation, invasion, and metastasis	miR-191, β-catenin	(93)
DSCR8	oncogene	Promote proliferation, and inhibit apoptosis	miR-485-5p, FZD7, β-catenin	(94)
FOXD2-AS1	oncogene	Promote proliferation and invasion	EMT, E2H2, DKK1, FOXA2, E-cadherin, MMP9, Cyclin D1, and c-Myc	(95)
CASC2c	suppressor	Inhibit proliferation and invasion	β-catenin	(97)
SUMO1P3	oncogene	Promote proliferation, invasion, and migration	miR-320a, β-catenin	(98)
CASC15	oncogene	Promote invasion	SOX4, β-catenin, Cyclin D1, and c-Myc	(99)
OTUD6B-AS1	oncogene	Promote proliferation and invasion	GSKIP, miR-664b-3p, β-catenin	(100)
LINC01278	oncogene	Promote invasion, migration	miR-1258, TCF-4, Smad2 and Smad3, β-catenin	(101)
LINC00662	oncogene	Promote proliferation migration	wnt3a, miR-15a, miR-16, and miR-107, β-catenin	(102)
DGCR5	suppressor	Promote invasion and migration	β‐catenin and cyclin D1, β-catenin	(103)
CCAL	oncogene	Promote proliferation and invasion	AP-2α, β-catenin	(104)

## Development of Cancer Stem Cells (CSCs)

**Figure 2:**
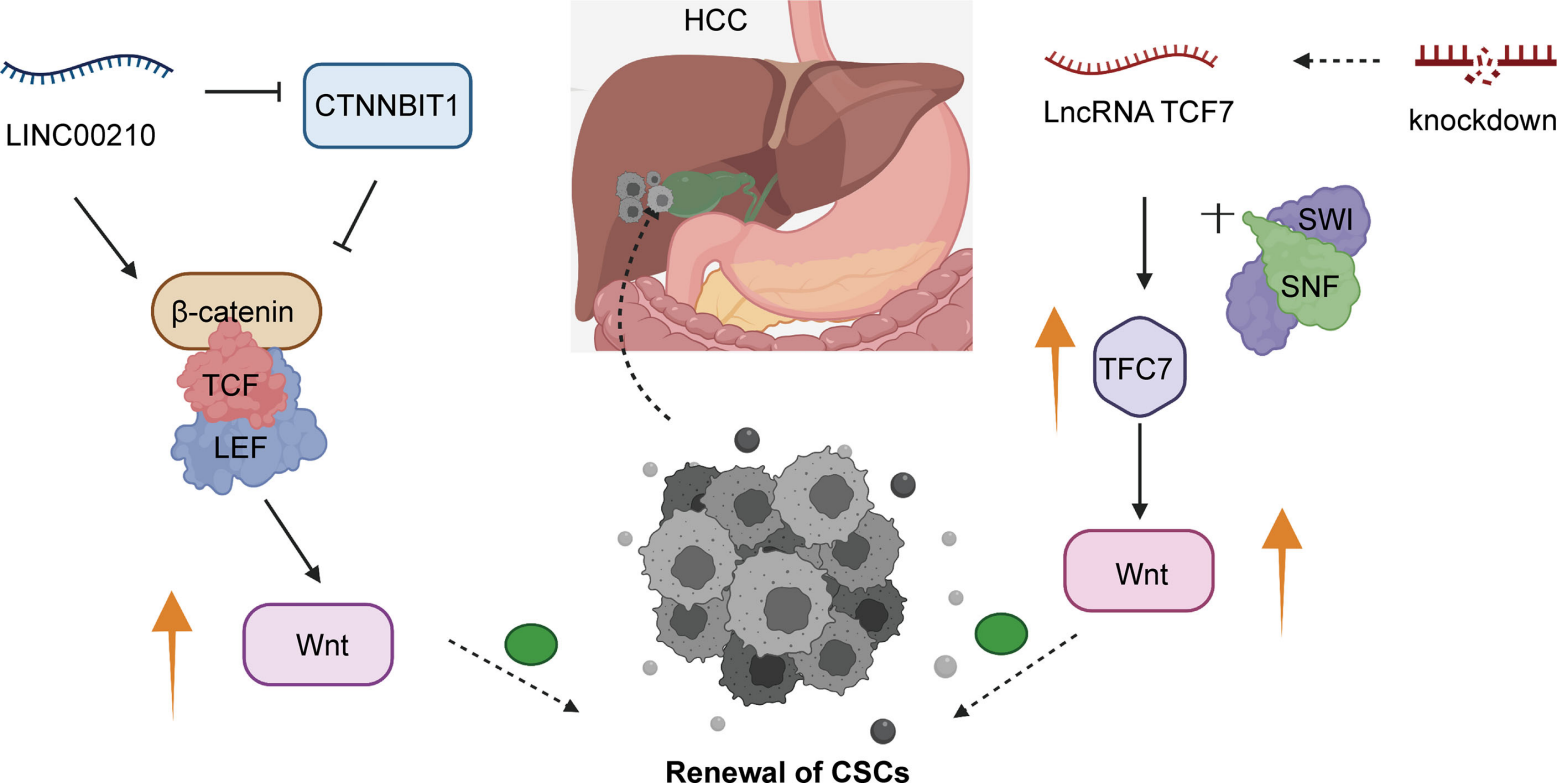
The specific mechanism of lncRNAs and the Wnt/β-catenin pathway in the development of HCC CSCs. Overexpression of linc00210 promotes the expression of β-catenin and the TCF/LEF complex to activate the Wnt/β-catenin pathway. lncTCF7 downregulation could impair the self-renewal ability of TICs in HCC.

CSCs were first verified in the xenotransplant process *via* the injection of acute myeloid leukemia (AML) cells into SCID mice, and their biological features resemble those of normal stem cells with self-renewal and differentiation abilities (155). The CSCs of HCC are termed liver cancer stem cells (LCSCs) or liver tumor-initiating cells (TICs), which have a crucial impact on tumor initiation, migration, and recurrence (156–158). Elevated expression of Linc00210 has been discovered in liver cancer and TICs. Emerging surface markers have been discovered to further identify TICs; these include CD13, CD133, CD24, and EPCAM (159–161). As a study reported, Wnt/β-catenin signaling is one of the pathways involved in liver cancer and is active in liver TICs (162). Linc00210 alleviates the suppressive role of *CTNNBIP1*, actively facilitating the binding of β-catenin and the TCF/LEF complex to trigger Wnt cascades. Furthermore, massive amplifications of linc00210 were shown to be vital for activating Wnt/β-catenin and stimulating TIC self-renewal (89). Likewise, LncTCF7 actively regulates TCF7 expression, primarily by recruiting the SWI/SNF complex. TCF7 upregulation remarkably increases the expression of Wnt signaling, inducing the initiation of self-renewal of TICs (89, 90). When TCF7 is expressed at low levels and the levels of Wnt downstream targets are reduced, the self-renewal capacity of TICs and tumorigenesis are rapidly attenuated in HCC. CSCs are similar to tissue-specific stem cells and control cell growth to significantly affect malignancy and tumor stage. TCF7 acts as an upstream activator of Wnt signaling to initiate the pathway (163, 164). Thus, overexpression of TCF7, which triggers the Wnt pathway, is required for the self-renewal of TICs and contributes to the promotion of HCC development. TCF7 was reported to participate in the tumorigenesis and progression of HCC (90) (**Figure 2**). On the basis of these findings that LncTCF7 impacts the biological processes of HCC *via* the TCF7/Wnt/β-catenin axis, it could be a promising therapeutic target to effectively improve the prognosis of HCC patients.

## Tumorigenesis

In HCC, lncRNA PRR34-AS1 was found to present increased expression and is involved in the promotion of tumorigenesis. Inhibition of miR-296-5p could positively mediate cell proliferation in HCC. E2F2 and SOX12 are considered essential downstream targets of miR-296-5p. A previous study found that E2F2 plays a significant role in regulating PRR34-AS1 transcription, contributing to enhanced expression of PRR34-AS1 in HCC cells (91). PRR34-AS1 could drive the Wnt pathway *via* the upregulation of SOX12. PRR34-AS1 is highly expressed in HCC and promotes tumorigenesis, and this effect is closely related to the miR-296-5p/E2F2/SOX12 axis. Additionally, E2H2, an epigenetic regulator, participates in the process of tumorigenesis (165). In most HCC patients, E2H2 is highly expressed and acts as a vital oncogene (166, 167). The expression of H3K27me3 (histone H3 lysine 27 trimethylation) was elevated by silencing Wnt pathway suppressors from E2H2, presenting a positive association with development in HCC. These inhibiting factors of Wnt signaling mainly include Axin2, DKK1 and Prickle1 (168). LncRNA H19 exerts a pivotal effect on hepatocarcinogenesis by mediating the EZH2/Wnt/β-catenin signaling axis (169). A recent study confirmed that the combination of F2C and curcumin obviously blocks tumor initiation in HCC. Moreover, marked downregulation of E2H2, β-catenin, and H3K27me3 was observed in the F2C-treated group. However, Axin2, DKK1 and Prickle1 were upregulated in the F2C-treated group (92). Further exploration revealed that F2C could suppress the expression of EZH2-H19 and promote the upregulation of Wnt signaling inhibitors, resulting in the blockade of tumorigenicity in HCC.

## Proliferation, Invasion, Metastasis

The rapid proliferation and high aggressiveness of cancer cells might commonly indicate the tendency of most patients to exhibit dismal outcomes. Several experiments revealed that knockdown of lncRNA ANRIL significantly inhibited the proliferation, invasion, and metastasis of HCC cells but induced apoptosis (170). ANRIL silencing observably reduced the expression of miR-191 in HCC cells. When miR-191 was overexpressed, the effect of ANRIL knockdown was attenuated to some extent. In diverse studies, miRNA-191 is regarded as an oncogene related to the aggressiveness of HCC cells (146, 171). ANRIL silencing could modulate miR-191 expression to impede the upregulation of the NF-κB and Wnt/β-catenin pathways (93). The upregulation of down syndrome critical region 8 (DSCR8) was confirmed to have a vital impact on the promotion of cell proliferation and the cell cycle and the restriction of apoptosis. Knockdown of DSCR8 was found to reverse the corresponding effect (94). The specific mechanism of DSCR8 has been continually explored, and its role in sponging miR-485-5p in HCC cells has also emerged. Unlike DSCR8, miR-485-5p was downregulated in HCC tissues. It was reported that FZD7 functions as the essential receptor driving the Wnt/β-catenin cascade (172) and acts as an oncogene in HCC (57). MiR-485-5p was found to decrease FZD7 expression, increase β-catenin expression in the nucleus and cytoplasm, and increase c-Myc and cyclin D1 expression in HCC cells. Cyclin D1 and c-Myc are known to be downstream of Wnt/β-catenin signaling. Consequently, lncRNA DSCR8 might positively affect the activation of Wnt/β-catenin to accelerate the growth of HCC cells *via* the instrumental DSCR8/miR-485-5p/FZD7 axis (94).

**Figure 3:**
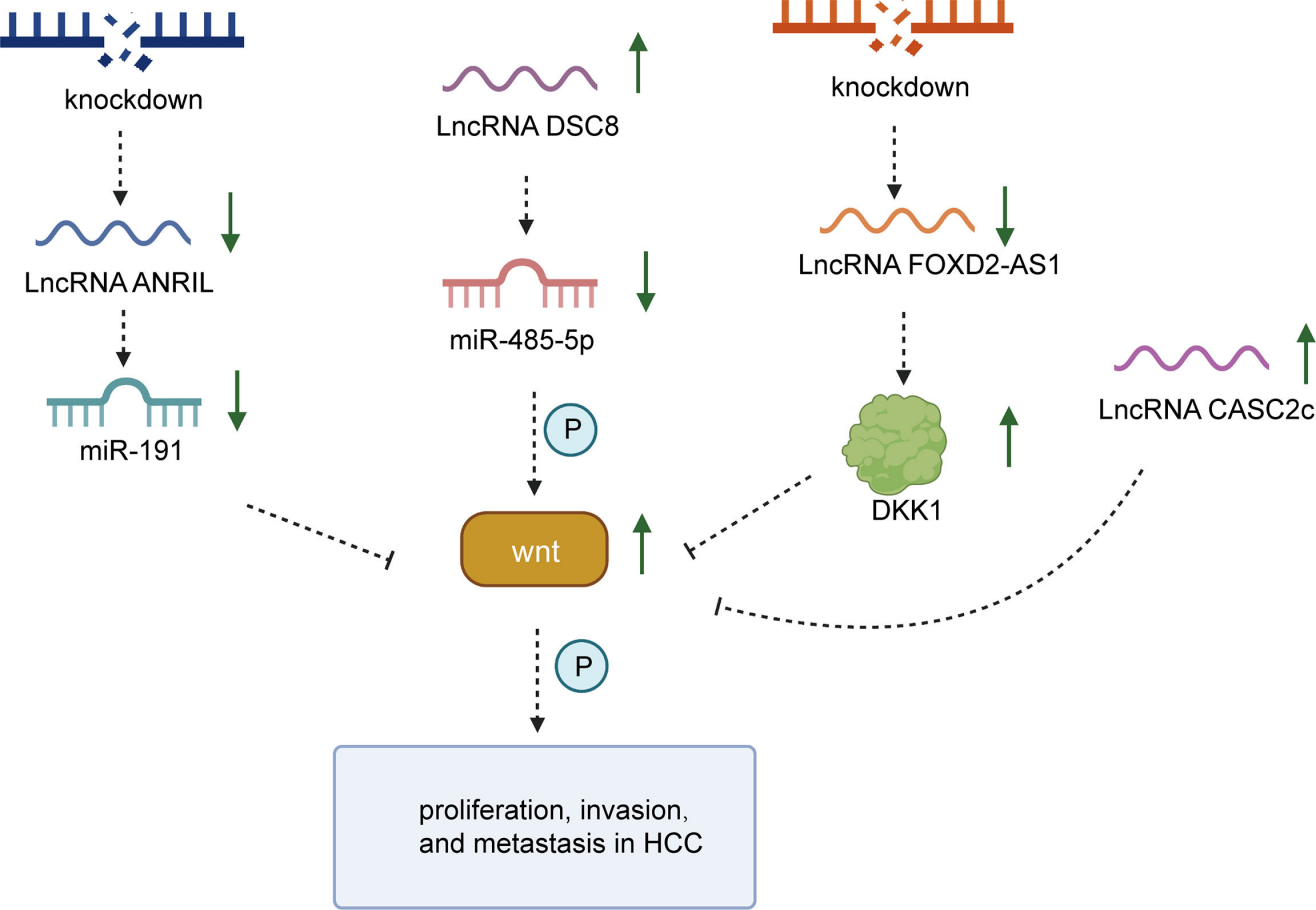
The specific mechanism of lncRNAs and the Wnt/β-catenin pathway in the proliferation, invasion and migration processes of HCC. LncRNA DSCR8 could sponge miR-485-5p to trigger Wnt/β-catenin signaling. Silencing of lncRNA ANRIL could block the progression of HCC by downregulating miRNA-191 to inhibit Wnt/β-catenin. Knockdown of lncRNA FOXD2-AS1 exerts a suppressive effect on the development of HCC *via* the inhibition of Wnt/β-catenin. Upregulation of CASC2c attenuates the activation of Wnt/β-catenin.

FOXD2-AS1 overexpression was proven to predict a poor prognosis of patients with HCC. In contrast, knockdown of FOXD2-AS1 might exert a negative effect on proliferation, invasion, and EMT progression. EGR1 can promote FOXD2-AS1 expression at the transcriptional level by binding with an activator of FOXD2-AS1. The effect of FOXD2-AS1 on promoting proliferation, invasion, and EMT was proven to positively mediate the Wnt/β-catenin signaling pathway. The mechanism by which FOXD2-AS1 interacts with the Wnt/β-catenin pathway was further explored. FOXD2-AS1 might regulate targets by binding with EZH2, specifically resulting in the epigenetic silencing of downstream factors. EZH2 could lead to a reduction in the expression of DKK1. FOXD2-AS1 is considered a key regulator that enhances the expression of Wnt/β-catenin signaling by silencing DKK1 (95). Liang et al. (96) showed that lncRNA-NEF could induce the phosphorylation of β-catenin to suppress the Wnt/β-catenin signal cascade, subsequently driving FOXA2 expression to suppress HCC metastasis. Accumulating evidence has revealed that FOXA2 can suppress EMT to hinder the migration of liver cancer through the modulation of E-cadherin and MMP9 expression (173). However, recent research revealed that CASC2c tends to be expressed at low levels in HCC tissues and cells, while CASC2c expression strongly blocks proliferation and reduces aggressiveness but induces apoptosis in HCC. CASC2c is regarded as a critical regulator that mediates ERK1/2 and Wnt/β-catenin signaling, and high expression of CASC2c attenuates ERK1/2 and Wnt/β-catenin cascades (97). Thus, CASC2c upregulation inhibits the activation of ERK1/2 and the Wnt/β-catenin pathway to promote apoptosis and suppress invasion and proliferation. Despite the inhibitory role of CASC2c in HCC, many more experiments are needed to clarify the complex mechanism (**Figure 3**).

## Clinical Applications of lncRNA and Wnt/β-Catenin Signaling in HCC

Overall, we elaborated the critical role of lncRNAs and the Wnt/β-catenin cascade in HCC to seek more hopeful and practical clinical applications to patients with HCC. Therefore, we further focused on diagnostic biomarkers, prognostic biomarkers and therapeutic targets of lncRNA and Wnt/β-catenin signaling.

## Effective Diagnostic Biomarkers

Owing to the lack of characteristic symptoms in the early stages of HCC, most patients are diagnosed in advanced stages with short survival times. Thus, experts have explored more effective diagnostic biomarkers of HCC in recent years (174–176). It was reported that increasing expression of LINC00355:8 played the oncogenic role in proliferation, invasion and metastasis ability of HCC. Upregulated LINC00355:8 mediated miR-6777-3p expression by acting as the ceRNA, giving rising to activation of Wnt/β-catenin signaling (113). In addition, upregulation of lncRNA CASC15 was observed in HCC, which suggests its positive associations with larger tumor size, higher tumor stage, and early lymph node metastasis (99). Silencing of CASC15 suppressed HCC development. SOX4 is regarded as the downstream target of CASC15, and its increased expression has been proven to attenuate the effect of inhibiting CASC15. The upregulation of SOX4 by CASC15 was found to have a major impact on the Wnt/β-catenin cascade. LncRNA RUNX1-IT1 (the intronic transcript 1 of RUNX1) was reported to express lowly in HCC patients correlated with shorter survival time. Mechanistically, downregulated LncRNA RUNX1-IT1 could simultaneously mitigate MiR-632 and GSK-3β expression to cause restoration of β-catenin, remaining CSC property and promoting invasion and metastasis in HCC (162). With regard to the diagnostic roles of lncRNAs in HCC, more research is required.

## Potent Prognosis Predictors

Effective prognostic biomarkers have become an indispensable part of clinical estimates of recurrence probability in HCC. Kong et al. (100) found that overexpression of OTUD6B-AS1 in HCC tissues was generally related to poor outcomes for patients, which indicated its potential for prognosis prediction. When OTUD6B-AS1 was silenced in HCC, cell proliferation, invasion, and colony formation were rapidly restricted (100). LncRNA Small nucleolar RNA host gene 5 (SNHG5) includes six exons and two snoRNAs (177), competitively binding with miR-26a-5p to enhance activity of Wnt/β-catenin pathway, which advances hepatocellular carcinoma progression (106). Thus, high-expressed lncRNA SNHG5 have strong association with poor prognosis HCC patients. Although LINC01278 was downregulated in papillary thyroid carcinoma, it is expressed at high levels in HCC tissues. LINC01278 upregulation is frequently positively correlated with worse prognosis. When LINC01278 is expressed at low levels, the invasion and migration in HCC is weakened by alleviating the induction of β-catenin and TGF-β1 (101). These results provide numerous markers to precisely assess the recurrence probability of patients with HCC.

## Practical Therapeutic Targets

Targeted treatment has recently received broad attention from experts due to its potency and effectiveness in therapeutic methods of multiple cancers (178–180). LINC00662 is overexpressed in HCC and positively associated with tumor size, malignant behaviors and worse prognosis. It was reported that LINC00662 can interact with miR-15a, miR-16, and miR-107 to promote Wnt3a upregulation, triggering Wnt/β-catenin cascades in an autocrine manner. Therefore, the effect of advancing tumor growth and migration induced by LINC00662 might be related to activation of the Wnt pathway (102). Liu et al. (104) validated that lncRNA CCAL is overexpressed in HCC tissues and correlated with tumor size and migration. Decreased expression of CCAL was found to weaken the invasiveness of HCC. Mechanistically, downregulation of CCAL could reduce AP-2α expression and suppress Wnt/β-catenin signaling (104). Elevated expression of lncRNA DLGAP1-AS1 (discs, large homolog-associated protein 1 antisense RNA 1) could downregulate miR-26a-5p and miR-26b-5p to participate in tumorigenesis process of HCC (114). Inversely, silencing of DLGAP1-AS1 play the suppressive role in HCC. Inhibitors of miR-26a/b-5p might recover knockdown of DLGAP1-AS1 by stimulating activity of Wnt/β-catenin pathway. Therefore, lncRNA DLGAP1-AS1 might become an effective target for therapy of HCC patients and prolonging survival time. These findings offer hope for the development of clinical treatments in HCC.

## Conclusions and Future Perspectives

The strikingly high incidence and mortality rates of HCC present threats to patient health and quality of life worldwide. The poor prognosis and rapid recurrence of HCC indicate the urgency of these studies and explorations on emerging methods for effective treatment. Fortunately, accumulating evidence has found that the interaction of lncRNAs and Wnt/β-catenin signaling provides a novel direction for understanding the pathogenesis of HCC that could improve clinical treatment and prolong survival time.

The Wnt/β-catenin pathway was proven to have a positive association with the EMT process. EMT is known as a regulator involved in the tumorigenesis and progression of several tumors. Thus, triggering the Wnt/β-catenin signaling pathway plays a vital role in the complicated mechanisms of various cancers. The degradation and accumulation of β-catenin have a great impact on the activation of the Wnt/β-catenin pathway. Recently, diverse lncRNAs have been demonstrated to participate in regulating β-catenin stability by affecting the formation of degradation complexes or regulating the transcriptional expression of β-catenin. Mechanistically, a wide range of lncRNAs are capable of functioning as ceRNAs to sponge targeted miRNAs, downstream effectors of lncRNAs, resulting in aberrant activation of the Wnt signaling pathway. Analogous mechanisms have also been identified in other malignant tumors and were found to be strongly related to the processes of cell proliferation, invasion, and metastasis.

The main focus of this review was to summarize most lncRNAs participating in the initiation and progression of hepatocellular carcinoma *via* the lncRNA/Wnt/β-catenin axis. These discoveries can be conducive to the identification of prospective molecular targets used for the effective treatment of HCC. However, several challenges have emerged in the application of clinical treatment. For instance, the upstream regulators of Wnt/β-catenin pathway activation vary, which suggests that other mechanisms and models affecting the pathogenesis of HCC remain unreported. Similarly, the structure and function of most lncRNAs are uncharacterized, which is an obstacle in research. Therefore, more research on lncRNAs related to Wnt/β-catenin and HCC is still needed.

## Author Contributions

All of the authors worked collaboratively on the work presented here. YH and WG designed the review. YX and XY wrote this manuscript. ZS searched the articles and made figures. All authors read and approved the final manuscript.

## Funding

This work was supported by the National Natural Science Foundation of China (81902832), the Youth Talent Lifting Project of Henan Province (2021HYTP059), and Key Scientific Research Project of Henan Higher Education Institutions ofChina (21A320026). This work was also supported by Leading Talents of Zhongyuan Science and Technology Innovation (214200510027), Henan Provincial Medical Science and Technology Research Plan (SBGJ202102117 and SBGJ2018002), Henan Medical Science and Technology Joint Building Program (LHGJ20210324), Science and Technology Innovation Talents in Henan Universities (19HASTIT003), Outstanding Foreign Scientist Studio in Henan Province (GZS2020004), and the Gandan Xiangzhao Research Fund (GDXZ2022002).

## Conflict of Interest

The authors declare that the research was conducted in the absence of any commercial or financial relationships that could be construed as a potential conflict of interest.

## Publisher’s Note

All claims expressed in this article are solely those of the authors and do not necessarily represent those of their affiliated organizations, or those of the publisher, the editors and the reviewers. Any product that may be evaluated in this article, or claim that may be made by its manufacturer, is not guaranteed or endorsed by the publisher.
